# Integrated Exposure-Based Therapy for Co-Occurring Post Traumatic Stress Disorder (PTSD) and Substance Dependence: Predictors of Change in PTSD Symptom Severity

**DOI:** 10.3390/jcm5110101

**Published:** 2016-11-15

**Authors:** Katherine L. Mills, Emma L. Barrett, Sabine Merz, Julia Rosenfeld, Philippa L. Ewer, Claudia Sannibale, Amanda L. Baker, Sally Hopwood, Sudie E. Back, Kathleen T. Brady, Maree Teesson

**Affiliations:** 1 National Drug & Alcohol Research Centre, University of New South Wales, Sydney 2052, NSW, Australia; e.barrett@unsw.edu.au (E.L.B.); sabinemerz@hotmail.com (S.M.); juliarosenfeld@hotmail.com (J.R.); philippa.ewer@gmail.com (P.L.E.); claudia.sannibale@exemail.com.au (C.S.); m.teesson@unsw.edu.au (M.T.); 2 Centre of Research Excellence in Mental Health and Substance Use, University of New South Wales, Sydney 2052, NSW, Australia; 3 Department of Psychiatry and Behavioral Sciences, Medical University of South Carolina, Charleston, SC 29425, USA; backs@musc.edu (S.E.B.); bradyk@musc.edu (K.T.B.); 4 School of Medicine and Public Health, University of Newcastle, Callaghan 2308, NSW, Australia; Amanda.Baker@newcastle.edu.au; 5 School of Psychology, University of New South Wales, Sydney 2052, NSW, Australia; s.hopwood@student.unsw.edu.au

**Keywords:** post traumatic stress disorder, comorbidity, psychological treatment

## Abstract

This paper examines factors associated with change in PTSD symptom severity among individuals randomised to receive an integrated exposure-based psychotherapy for PTSD and substance dependence–Concurrent Treatment of PTSD and Substance Use Disorders Using Prolonged Exposure (COPE). Outcomes examined include change in PTSD symptom severity as measured by the Clinician Administered PTSD Scale (CAPS), and the reliability and clinical significance of change in PTSD symptom severity. Factors examined include patient baseline characteristics, treatment characteristics, and events over follow-up. The mean difference in CAPS score was 38.24 (SE 4.81). Approximately half (49.1%) demonstrated a reliable and clinically significant improvement in PTSD symptom severity. No one was classified as having demonstrated clinically significant worsening of symptoms. Three independent predictors of reductions in PTSD symptom severity were identified: baseline PTSD symptom severity (β 0.77, *SE* 0.23, *p* = 0.001), number of traumas experienced prior to baseline (β −0.30, *SE* 0.15, *p* = 0.049), and number of sessions attended (β 2.05, *SE* 0.87, *p* = 0.024). The present study provides further evidence regarding the safety of the COPE treatment and factors associated with improvement in PTSD symptom severity. The identification of only a small number of predictors of the outcome points to the broad applicability of the COPE treatment to PTSD and substance use disorder (SUD) patients.

## 1. Introduction

The high prevalence and considerable harm associated with post-traumatic stress disorder (PTSD) among people with substance use disorders (SUD) has been well documented. Up to two-thirds of people entering SUD treatment settings have been found to meet the criteria for a diagnosis of PTSD [1,2,3,4,5], which is associated with a more severe clinical profile characterised by an earlier age of onset of substance use, more extensive polydrug use histories, poorer educational and occupational functioning, poorer physical health, greater psychopathology, and higher rates of overdose and attempted suicide [2,6,7,8,9,10,11,12,13,14,15]. Given this clinical profile, it is not surprising that PTSD has been associated with poorer outcomes across a number of domains, particularly with regard to general physical and mental health and psychosocial functioning [16,17]. Research examining the relationship between SUD and PTSD has also highlighted the importance of treating PTSD in order to improve long-term substance use outcomes [12,18,19]. Collectively, this research has led to numerous position papers, policy documents, and clinical guidelines, internationally, that call for the provision of integrated treatment for individuals with comorbid PTSD and SUD; that is, treatment of both disorders at the same time by the same therapist [20,21]. Many patients with comorbid PTSD and SUD also indicate that they would prefer to receive integrated treatment [18,22,23].

Research examining the efficacy of integrated treatments has predominantly focused on present-centred, cognitive-behavioural therapies that focus on teaching safe coping skills relevant to both trauma and substance use to deal with present day problems, without revisiting the traumatic memory [24,25,26]. More recently, research has emerged examining the efficacy of cognitive-behavioural therapies that incorporate exposure-based techniques. The core feature of these approaches is prolonged and repeated exposure to memories (imaginal exposure) and physical reminders (in vivo exposure) of past trauma. Exposure therapy has long been regarded as a gold standard treatment, alongside eye movement desensitization and cognitive reprocessing therapy [21,27]. However, until recently, it was considered to be contraindicated in patients with SUD [28] because it was widely believed that the intense emotions elicited during exposure could place individuals at increased risk for relapse [29,30]. It was also thought that substance use would impair fear activation and processing of new information, thereby reducing treatment effectiveness, and that cognitive impairment associated with SUD may impair patients’ ability to undertake imaginal exposure [31]. Consequently, patients with SUD have been excluded from most trials of prolonged exposure [28]. Findings from a growing number of studies are, however, challenging these assumptions. Support for integrated treatments that incorporate exposure techniques is growing with a number of studies providing evidence for their safety and efficacy [32,33,34,35,36,37].

In 2012, we published the first randomised controlled trial to examine the efficacy of an integrated treatment for PTSD and substance dependence that incorporates prolonged exposure therapy [33] called Concurrent Treatment of PTSD and Substance Use Disorders Using Prolonged Exposure (COPE, [38,39]). Compared to individuals randomised to treatment-as-usual (TAU) for their substance use alone, individuals randomised to receive COPE plus TAU (COPE + TAU) demonstrated significantly greater reductions in PTSD symptom severity (a 16.09 difference in change scores on the Clinician Administered PTSD Scale (CAPS), and were less likely to meet criteria for a diagnosis of PTSD at nine-month follow-up (79.2% vs. 56.4%). In the present paper, we build on this research by examining factors associated with change in PTSD symptom severity among those randomised to receive COPE. More specifically, we examine the impact of patient characteristics at treatment entry, treatment characteristics, and events over follow-up. This knowledge is essential to improving our understanding of which patients, and under what circumstances, this treatment works best.

## 2. Methods

### 2.1. Procedure

Methods for this study have been described in detail elsewhere [33]. In brief, 103 participants (83.1% response rate) were recruited between April 2007 and June 2009 from substance use treatment services, media advertisements, and practitioner referrals within the greater Sydney region, Australia. Participants were randomly assigned to receive either (i) COPE+TAU (*n* = 55); or (ii) TAU for substance use alone (*n* = 48). The present paper focuses only on the 55 individuals randomised to the COPE+TAU intervention.

Inclusion criteria were: (i) met criteria for past-month DSM-IV-TR diagnoses of PTSD and substance dependence; ii) aged 18 years or over; and (iii) fluent in English. Exclusion criteria were (i) currently suicidal (expressed suicidal ideation accompanied by a plan and intent); (ii) recent history of self-harm (past six months); (iii) current active symptoms of psychosis; or (iv) cognitive impairment severe enough to impede treatment. Outcomes were assessed at nine-months post-baseline, and interim measures collected at six-weeks and three-months post-baseline. Thirty-nine participants were re-interviewed at nine-month post-baseline (70.9%). Ethical approval was granted by the Human Ethics Review Committees of the University of New South Wales and the Northern Sydney Central Coast Area Health Service.

### 2.2. Structured Interviews

All participants were administered a structured, face-to-face interview at baseline. PTSD symptom severity was assessed using the Clinician-Administered PTSD Scale (CAPS) [40]. The interview also assessed demographic characteristics; lifetime and current drug use (heroin, other opiates, amphetamines, cocaine, hallucinogens, benzodiazepines, alcohol, cannabis, and inhalants) using the Opiate Treatment Index [41]; severity of dependence (as indicated by the number of dependence criteria met) and DSM-IV-TR diagnoses of current dependence using the Composite International Diagnostic Interview (CIDI) version 3.0 [42]; trauma history using the CIDI version 2.1 [43]; depression using the Beck Depression Inventory-II (BDI-II) [44]; state and trait anxiety using the State-Trait Anxiety Inventory (STAI) [45]; the possible presence of borderline personality disorder (BPD) using the International Personality Disorder Examination Questionnaire [46]; and history of attempted suicide. To assess substance use treatment history participants were asked whether they had commenced any of the following: substitution pharmacotherapies (including methadone, buprenorphine, Suboxone, and naltrexone maintenance); outpatient or inpatient detoxification; residential rehabilitation; and outpatient counselling. To assess PTSD treatment history, participants were asked whether they had ever commenced any of the following for their PTSD: inpatient hospitalisation; outpatient counselling or psychotherapy, and medication (such as antidepressants).

The sections of the assessment pertaining to current drug use, dependence, PTSD, depression, and anxiety were re-administered at each follow-up interview. Participants were also asked whether they had been exposed to any further traumatic events, had experienced any suicidal ideation or attempted suicide, or undergone any treatment over the follow-up period. The number of days in treatment was calculated and divided by the number of days to follow-up to give the proportion of time spent in treatment over the follow-up period.

Participants were paid AUD$30 for completing each interview. Interviews were administered by two trained research officers blind to group allocation.

### 2.3. Interventions

#### 2.3.1. COPE

COPE is a modified version of Concurrent Treatment of PTSD and Cocaine Dependence [47]. The version of COPE used in the present study represents an integration of existing evidence-based, manualized CBT interventions for PTSD and SUD [48,49,50]. The intervention consists of 13 individual 90-minute sessions (i.e., 19.5 h) delivered by a clinical psychologist and combines CBT for PTSD and SUD. Although designed to be delivered weekly, flexibility is permitted. Treatment components include: motivational enhancement and CBT for SUD (Sessions 1–4 and throughout); psychoeducation relating to both disorders and their interaction (Sessions 1–4); in vivo exposure (Sessions 5–12); imaginal exposure (Sessions 6–12); and cognitive therapy for PTSD (Sessions 8–12). The final session (Session 13) is dedicated to providing a review of the treatment, devising an after care plan, and termination.

COPE was delivered by two female clinical psychologists employed on the project who received fortnightly supervision for the duration of the study. All treatment sessions were recorded. Ten percent of participants were randomly selected to have their sessions rated for treatment fidelity (i.e., compliance with the treatment manual) by an independent assessor. Fidelity was rated on 53 (16.4%) out of a total of 323 sessions conducted as part of the study. Average fidelity ratings were high with a mean score of 4.13 (SD 0.95) out of a possible score of 5 indicating strong adherence to the treatment manual.

#### 2.3.2. TAU

Both the treatment and control groups were able to engage in TAU for SUD. As such, participants could access any type of SUD treatment currently available in the community, including outpatient counselling, inpatient or outpatient detoxification, residential rehabilitation and pharmacotherapies (e.g., methadone, buprenorphine, Suboxone, naltrexone).

### 2.4. Statistical Analyses

As described elsewhere [33], to satisfy the intention-to-treat requirement that analyses be undertaken on all participants, missing data were imputed using multiple imputation, which allows for the uncertainty about the missing data by creating several different plausible imputed datasets and appropriately combining results obtained from each. Multiple imputation is recommended over single-imputation techniques because the missing values for each participant are predicted from his or her own observed values, and the estimates produced take into account the uncertainty of the imputation process.

Two-sided analyses were conducted with IBM SPSS Statistics for Windows, Version 20.0. (Armonk, NY, USA) using a predetermined alpha level of *p* < 0.05. Outcomes examined were: (i) change in PTSD symptom severity; and (ii) the reliability and clinical significance of change in PTSD symptom severity.

Change in PTSD symptom severity was calculated by subtracting participants’ follow-up CAPS score from their baseline CAPS score. Thus, positive values are indicative of improvement and negative values indicative of worsening. The reliability and clinical significance of the changes were assessed using criteria established by Jacobson and Truax [51]. Specifically, reliable change index (RCI) scores was derived by dividing the difference between the observed post-test ($x_{post}$) and pre-test ($x_{pre}$) scores by the standard error of the differences:

(1) $$RCI=\;\frac{\left(x_{post}-x_{pre}\right)}{\sqrt{2S_{E}^{2}}}$$

Calculation of the standard error ($S_{E}$) was dependent on the reliability of the outcome measure (r), which was defined as the correlation between pre- and post-test CAPS scores:

(2) $$S_{E}=SD\sqrt{1-r}$$

RCI scores were then used to classify participants as having demonstrated reliable and clinically significant (i) improvement (RCI < −1.96); (ii) worsening (RCI ≥ 1.96), or no change (RCI > −1.96 and <1.96), in PTSD symptom severity.

A series of linear and logistic regression analyses were undertaken to determine factors associated with outcome. Factors examined include participant baseline characteristics (demographics, substance use, trauma history, mental health), COPE treatment characteristics (attendance, retention, time taken to deliver, therapist, receipt of imaginal and in vivo exposure), and events over follow-up (i.e., substance use treatment and trauma exposure). All models controlled for baseline PTSD symptom severity (i.e., baseline total CAPS score). Results are reported as the unstandardized mean difference with a 95% confidence intervals (95% CI) for linear models, and odds ratios (OR) with 95% CI for binomial logistic models.

## 3. Results

### 3.1. Participant Baseline Characteristics

#### 3.1.1. Demographics

As described in Mills et al. [33], the mean age of participants randomised to receive COPE was 33.4 years (SD 7.4) and 60% were female. Most were Australian born (85.5%); only 3.6% identified as being of Aboriginal or Torres Strait Islander origin. The median years of school completed was 10 (range 7–12) and 72.7% had completed tertiary education. Most were unemployed (76.4%) and 30.9% had a history of imprisonment.

#### 3.1.2. Substance Use

The median age of first intoxication was 13 years (range 7–29 years) and 78.2% had a history of injecting drug use. Poly-substance use was the norm, with participants using a median of 4.0 different drug classes in the preceding month (range 1–8), most commonly cannabis (74.5%), benzodiazepines (69.1%), and alcohol (67.3%), followed by amphetamines (41.8%), heroin (41.8%), other opiates (30.9%), cocaine (23.6%), hallucinogens (18.2%), and inhalants (3.6%). The most commonly reported main drug of concern was cannabis (23.6%), followed by heroin (20.0%), amphetamines (16.4%), benzodiazepines (16.4%), alcohol (12.7%), cocaine (9.1%), and other opiates (1.8%). Most had a history of prior substance use treatment (90.9%)

#### 3.1.3. Trauma History

Participants reported exposure to a wide range of trauma types (median 6, range 2–9) and all had experienced multiple traumas. The most common traumas experienced were physical assault (94.5%) and having been threatened or held captive (90.9%), followed by witnessing serious injury or death (83.6%), sexual assault (i.e., rape or molestation; 76.4%), experiencing a life threatening accident or natural disaster (72.7%), torture (27.3%), and combat (1.8%). Most (70.9%) also reported experiencing some “other” traumatic event. The median age of first trauma was 10 years (range 1–44); 69.1% experienced trauma during childhood (<16 years) and 45.5% had experienced childhood sexual abuse.

#### 3.1.4. Mental Health

All subjects met the DSM-IV diagnostic criteria for PTSD (25.5% delayed onset) and had experienced symptoms for a median of 9 years (range 0.25–36). Only on third (30.9%) had a history of prior PTSD treatment. The mean state and trait anxiety scores were 54.69 (SE 1.80) and 62.22 (SE 1.30) respectively, and the mean BDI score 36.07 (SE 1.49). More than two-thirds (69.1%) screened positive for BPD and 58.2% had ever attempted suicide, 10.9% in the previous 12 months.

### 3.2. COPE Treatment Characteristics

**Figure 1:**
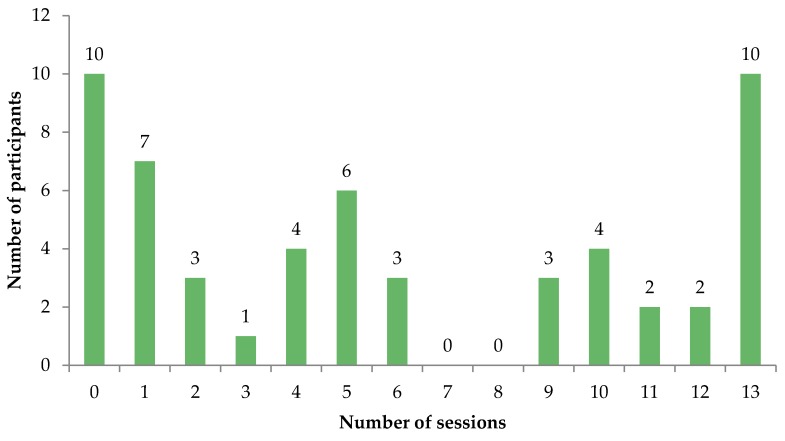
Total number of COPE sessions attended.

As shown in Figure 1, 45 participants (81.8%) attended at least one session (median 5, range 0–13) and 30 participants (54.5%) attended sessions in which imaginal or in vivo exposure were covered (40.0% imaginal, 50.9% in vivo). Participants attended a median of zero sessions that included imaginal exposure (range 0–7) and one covering in vivo exposure (median 0–8). Ten participants (18.2%) attended all 13 sessions.

The delivery of COPE treatment was spread evenly across the two therapists (49.1% and 50.9%). There was considerable variability between participants in the time taken to deliver the COPE treatment, ranging from 0–271 days (median 71 days). Twenty-two (40.0%) participants were still receiving COPE treatment after three months.

### 3.3. Events over Follow-Up

#### 3.3.1. Substance Use Treatment

Eighty percent were enrolled in TAU for their substance use at study entry, most commonly detoxification (50.9%), followed by maintenance therapies (21.8%) and residential rehabilitation (7.3%). Participants spent 54.7% (SE 5.52) of the follow-up period in TAU.

#### 3.3.2. New Trauma Exposure

Thirty-three participants (60.4%) experienced further trauma over the nine-month follow-up including witnessing injury or death (33.3%), physical attack (25.6%), being threatened or held captive (17.9%), sexual assault (12.8%), some “other” traumatic event (12.8%), great shock due to a traumatic event occurring to a loved one (12.8%), life threatening accident (7.7%), and natural disaster (2.6%).

### 3.4. Change in PTSD Symptom Severity

The mean difference in CAPS score between baseline and nine-month follow-up was 38.24 (SE 4.81). Approximately half (49.1%) demonstrated a clinically significant improvement in PTSD symptom severity and 50.9% demonstrated no change. No one was classified and having demonstrated clinically significant worsening of symptoms.

### 3.5. Characteristics Associated with Change in PTSD Symptom Severity

#### 3.5.1. Participant Characteristics

Higher PTSD symptom severity at baseline was positively associated with reductions in PTSD symptom severity over follow-up (Table 1). After controlling for baseline PTSD symptom severity, the only other participant characteristic positively associated with reductions in PTSD symptom severity was having completed a greater number of school years. No significant differences were found in relation to other demographic characteristics, substance use, mental health, or treatment; however, the number of trauma types experienced was negatively associated with reductions in PTSD symptom severity. There were no significant differences found between individuals who demonstrated clinically significant improvement in PTSD symptom severity and those who demonstrated no change.

#### 3.5.2. COPE Treatment Characteristics

After controlling for baseline PTSD symptom severity, reductions in PTSD symptom severity were positively associated with having started COPE therapy (i.e., attending at least one session), the number of sessions attended, and having undergone imaginal and/or in vivo exposure (Table 2). There were no significant differences found between individuals who demonstrated clinically significant improvement in PTSD symptom severity and those who demonstrated no change.

#### 3.5.3. Events over Follow-Up

After controlling for baseline PTSD symptom severity, proportion of time spent in SUD treatment, use of antidepressants, and exposure to new traumas over the follow-up period were not significantly associated with change in PTSD symptom severity (Table 2). There were no significant differences found between individuals who demonstrated clinically significant improvement in PTSD symptom severity and those who demonstrated no change.

### 3.6. Independent Predictors of Change in PTSD Symptom Severity

Participant and COPE treatment characteristics found to be significantly associated with change in PTSD symptom severity were entered into a multivariate linear regression. These included baseline PTSD symptom severity, years of school completed, number of traumas experienced prior to baseline, starting COPE therapy, and number of sessions attended. Imaginal and in vivo exposure were not included due to high correlation with the number of sessions attended (*r_pb_* = 0.89 and *r_pb_* = 0.72, respectively). Backward stepwise removal was utilised to determine independent predictors of change in PTSD symptom severity. Variables were removed from the model at *p* > 0.10. Three variables were removed over three steps. Variables retained in the final model were baseline PTSD symptom severity (β 0.77, *SE* 0.23, *p* = 0.001), number of traumas experienced prior to baseline (β −0.30, *SE* 0.15, *p* = 0.049), and number of sessions attended (β 2.05, *SE* 0.87, *p* = 0.024).

## 4. Discussion

This secondary analysis presents the first examination of predictors of change in PTSD symptom severity among people randomised to receive an integrated exposure-based treatment for PTSD and SUD. Participants randomised to receive COPE demonstrated significant reductions in PTSD symptom severity (38.24 points on the CAPS scale), with mean CAPS scores reducing from the extreme to moderate range [52]. These reductions in symptom severity were also accompanied by diagnostic shifts (43.6% reduction in PTSD diagnoses) [33]. Close to one-half (49.1%) were classified as having demonstrated reliable and clinically significant improvement. This rate of improvement is similar to that derived from a meta-analysis of 26 RCTs examining psychotherapies for PTSD more broadly (44%), the majority of which excluded individuals with SUD [53], but somewhat surprising given the severe clinical profile exhibited by participants at study entry. Most importantly, no participant was classified as having demonstrated reliable and clinically significant worsening of symptoms, providing further evidence in support of the safety of using integrated exposure-based approaches to treat PTSD among people with SUDs.

Although these findings are promising, 50.9% of those randomised to COPE did not demonstrate a reliable and clinically significant change in symptom severity, and 56.4% continued to meet diagnostic criteria for PTSD following treatment [33]. Research conducted by Schnurr and Lunney [54,55] among female veterans found that although a reduction of as little as 10 points on the CAPS is associated with clinically meaningful change, loss of diagnosis is necessary to optimise gains in quality of life and functioning.

It is possible that further improvements would have been obtained with greater retention or lengthier treatment. Indeed, number of sessions attended was one of only three variables found to independently predict change in PTSD symptom severity. It is important to note, however, that improvements in PTSD symptom severity among clients of substance use services have been observed following even very brief treatments [56]. Furthermore, even if all clients received a full dose of treatment, it is unlikely that all would improve.

In the present study, we were unable to separate the effects of having received the exposure-based components of the treatment from those attributable to the number of sessions attended, due to the high correlation between these variables. Previous research has however, identified that imaginal and in vivo exposure specifically appear to be play an important role in obtaining improvements in PTSD [57,58]. Furthermore, a recent Cochrane review of integrated psychotherapies for PTSD and SUD concluded that individual trauma-focused psychological intervention delivered alongside SUD intervention can reduce PTSD severity and substance use, but that there is very little evidence to support use of non-trauma-focused individual or group-based interventions [24]. As noted by the authors of this review, however, the evidence to date is limited and generally of low quality. Continued investigation of alternate treatment options for this comorbidity, both trauma-focused and non-trauma-focused, is needed, including investigation of stepped-care approaches.

There has long been concern that the distress associated with exposure-based therapies would be aversive to clients, leading to reduced treatment retention [29,59]; however, several studies have shown that this is not the case. Retention and dropout rates for exposure therapies have been found to be comparable to that of other non-exposure based psychotherapies for PTSD [60,61,62,63]. Furthermore, treatment retention in the present study is comparable with that found among integrated treatment of PTSD and SUD that is not trauma-focused [19]. Thus, further research examining methods to improve retention in treatment is needed for both trauma-focused and non-trauma-focused therapies. Efforts to enhance treatment engagement and therapeutic alliance may be of particular relevance to this patient population. For those who have experienced interpersonal trauma in particular, issues relating to trust and interpersonal connectedness, as well as the power imbalance inherent in a therapeutic relationship, may impact on a person’s ability and willingness to engage in treatment [64]. Provision of concurrent case management, and involvement of other health and social services to manage clients’ broader needs, may also be useful in increasing retention when provided within a coordinated care framework [65,66].

Research examining baseline patient characteristics as predictors of PTSD treatment outcome has produced inconsistent results [67,68]. In terms of individual patient characteristics, only two factors were found to independently predict change in PTSD symptom severity. Higher PTSD symptom severity at baseline was positively associated with reductions in PTSD symptom severity over follow-up, and the number of trauma types experienced was negatively associated with reductions in PTSD symptom severity. This finding is consistent with previous research [68], and may reflect the need for lengthier treatment among clients with complex trauma histories in order to address symptoms associated with different trauma types.

Importantly, change in PTSD symptom severity was not influenced by the presence of other comorbidities (i.e., depression, anxiety, BPD), types of trauma experienced, type or extensiveness of substance use, or treatment history. These findings add to the growing literature demonstrating that, contrary to popular belief, comorbidity should not be regarded as a universal contraindication to exposure-based therapies [28,68]. Similarly, experiencing further trauma, use of antidepressants and further substance use treatment did impact on outcome.

It is important to note that although two patient characteristics and one treatment characteristic were found to independently predict a change in PTSD symptom severity as a continuous measure, none of the characteristics examined were found to be associated with reliable and clinically significant change in PTSD symptom severity. The identification of only a small number of predictors of outcome points to the broad applicability of the COPE treatment to PTSD and SUD patients. It is however, also possible that the lack of associations found was due to the small sample size.

A number of other limitations should also be noted. Firstly, although the characteristics of the sample (i.e., participants who had experienced a wide range of traumas, were using a variety of substances, and experienced significant comorbidity) lend support to the generalizability of the findings, the findings cannot be generalised to individuals younger than 18 years; those not fluent in English; those currently suicidal, self-harming, or psychotic; or those with severe cognitive impairment, because these individuals were excluded from study participation. The sample size precluded a more detailed examination as to whether outcomes differed according to drug of concern. Future research with larger samples examining these associations would contribute significantly to our understanding of which clients benefit from this treatment. The finding that cannabis was the most frequently reported main drug of concern is not particularly surprising given that (i) it is one of the most prevalent illicit drug classes used, and for which people seek treatment, in Australia [69,70]); and (ii) cannabis has been found to relieve some PTSD symptoms and is legally prescribed for this purpose in some US states [71,72].

Secondly, the study relied on measures of self-report alone. There is debate regarding the reliability and validity of self-reported drug use; however, an extensive literature documents its reliability and validity [73]. Overall, agreement between self-report and biomarkers is high; indeed, when there are discrepancies, this tends to be when respondents report drug use that has failed to be detected by the biological measures. With regard to the analyses, it should also be noted that to satisfy the intention-to-treat requirement that outcome data be analysed for all participants, missing data were imputed. Although the methods used in the present study are considered optimal and take into account the uncertainty surrounding the imputation process, the actual values for missing participants remain unknown. The analyses were also based on a predetermined α level of *p* < 0.05, and adjustments were not made to take into account multiple comparisons.

## 5. Conclusions

In sum, the present study provides further evidence regarding the safety of the COPE treatment and factors associated with improvement in PTSD symptom severity. This knowledge is essential to improving our understanding of which patients, and under what circumstances, this integrated treatment works best. Despite exhibiting a severe clinical profile upon entry to the study, close to one-half demonstrated reliable and clinically significant improvement in PTSD symptom severity. No participant was classified as having demonstrated reliable and clinically significant worsening of symptoms. The study identified only three independent predictors of change in PTSD symptom severity, indicating the treatment is appropriate for a broad range of patients with PTSD and SUD.

## Figures and Tables

**Table 1:** Results of regression analyses examining associations between participant characteristics and change in PTSD symptom severity ^a^.

	Change in PTSD Symptom Severity			Clinically Significant Change in PTSD Symptom Severity		
	β	*SE*	*p*	Improvement (*n* = 27)	No change (*n* = 28)	OR (95% CI)
**Demographic characteristics**						
Age	0.21	0.52	0.691	33.64 (SE 1.51)	33.93 (SE 1.41)	1.00 (0.92–1.08)
Female sex	9.17	8.49	0.282	63.6	56.3	1.82 (0.54–0.61)
Born in Australia	6.44	12.02	0.593	90.0	80.7	3.53 (0.55–22.67)
Years of school completed	6.06	2.68	0.024	10.59 (0.24)	10.14 (0.31)	1.36 (0.81–2.26)
Unemployed	2.81	9.10	0.309	83.0	70.0	1.74 (0.44–6.86)
History of imprisonment	−15.82	10.32	0.137	18.5	42.9	0.37 (0.09–1.51)
**Substance use**						
Age of first intoxication	2.19	1.25	0.084	13.68 (0.77)	12.36 (0.53)	1.27 (0.98–1.65)
History of injecting drug use	7.04	9.44	0.456	28.1	15.7	1.63 (0.34–7.76)
Number of drug classes used (last month	−0.18	2.88	0.950	3.67 (0.31)	3.75 (0.31)	1.07 (0.69–1.66)
Main drug of concern alcohol	1.33	13.30	0.920	14.1	11.4	1.25 (0.16–9.53)
**Trauma and PTSD**						
Age of first trauma exposure	0.69	0.46	0.132	13.77 (1.82)	9.82 (1.52)	1.08 (0.99–1.17)
Age of worst trauma exposure	0.10	0.10	0.302	21.6 (2.15)	23.55 (2.37)	0.98 (0.93–1.03)
Childhood trauma	−7.69	9.28	0.410	60.7	77.1	0.39 (0.11–1.42)
Types of trauma						
Life threatening accident	−.312	9.30	0.973	65.2	69.3	0.82 (0.18–3.75)
Natural disaster	−10.04	9.32	0.283	22.2	32.1	0.66 (0.15–2.86)
Witness serious injury or death	−13.43	10.44	0.20	80.0	87.1	0.65 (0.13–3.2)
Sexual assault	−3.80	10.48	0.719	71.9	80.7	0.66 (0.13–3.21)
Physical assault	−13.43	16.82	0.424	96.3	92.9	2.05 (0.15–27.58)
Threatened	6.81	13.6	0.617	92.6	89.3	1.15 (0.15–8.65)
Torture	−7.15	9.22	0.439	28.9	25.7	.93 (0.25–3.49)
Other	12.26	8.84	0.170	41.5	31.4	1.53 (0.33–7.13)
Great shock	−2.11	8.31	0.799	57.8	51.4	1.54 (0.40–5.91)
Number of trauma types experienced	−2.70	2.71	0.323	6.02 (0.32)	6.23 (0.29)	0.95 (0.66–1.37)
Number of traumas experienced	−0.324	0.159	0.042	16.99 (3.50)	23.02 (5.43)	0.99 (0.96–1.02)
Trauma exposure preceded age of first intoxication	−7.02	8.51	0.412	44.4	64.3	0.37 (0.09–1.50)
Duration of PTSD symptoms	−0.064	0.41	0.709	11.85 (1.98)	8.57 (1.83)	1.04 (1.98–1.11)
Severity of PTSD symptoms	0.64	0.25	0.010	95.57 (3.01)	86.89 (2.91)	1.04 (1.00–1.08)
**Mental health**						
Severity of depression	−0.19	0.48	0.687	36.69 (2.42)	35.44 (2.07)	0.98 (0.90–1.06)
State anxiety	0.134	0.305	0.660	56.13 (2.99)	53.33 (2.48)	1.01 (0.96–1.06)
Trait anxiety	−0.025	0.495	0.960	61.76 (2.34)	62.62 (1.67)	0.97 (0.89–1.06)
Screened positive for BPD	−8.23	8.75	0.348	63.7	74.3	0.55 (0.12–2.43)
**Treatment history**						
History of SUD treatment	0.47	13.72	0.973	91.9	90.0	1.17 (0.13–10.28)
History of PTSD treatment	2.67	10.16	0.794	27.4	34.3	0.62 (0.13–2.96)
Engaged in current SUD treatment	−4.48	10.82	0.680	84.4	75.7	1.39 (0.27–7.10)
Engaged in current PTSD treatment	5.97	14.34	0.677	7.4	10.7	0.27 (0.03–2.37)
Current use of antidepressants	1.51	9.21	0.870	57.0	52.1	1.29 (0.32–5.21)

^a^ all models controlled for baseline PTSD symptom severity (i.e., total CAPS score).

**Table 2:** Results of regression analyses examining associations between treatment characteristics and events over follow-up and change in PTSD symptom severity ^a^.

	Change in PTSD Symptom Severity			Clinically Significant Change in PTSD Symptom Severity		
	β	*SE*	*p*	Improvement (*n* = 27)	No change (*n* = 28)	OR (95% CI)
**COPE treatment characteristics**						
Therapist	4.13	7.89	0.601	50.4	51.4	0.99 (0.29–3.40)
Started COPE therapy	22.76	11.04	0.044	94.8	69.3	–
Number of sessions attended	2.14	0.88	0.019	6.27 (0.91)	5.53 (1.14)	1.07 (0.92–1.25)
Received imaginal and/or in vivo exposure	19.75	8.81	0.031	60.7	48.6	2.26 (0.46–11.03)
**Events over follow-up**						
Proportion of time spent in SUD treatment	−4.26	11.08	0.701	0.51	0.57	0.90 (0.19–4.27)
Use of antidepressants	8.56	10.19	0.401	63.7	49.3	1.22 (0.28–5.29)
Trauma exposure over follow-up	−13.50	8.90	0.132	50.4	55.0	0.60 (0.16–2.28)

^a^ all models controlled for baseline PTSD symptom severity (i.e., total CAPS score).
